# *SLCO1B1* Genetic Variation Influence on Atorvastatin Systemic Exposure in Pediatric Hypercholesterolemia

**DOI:** 10.3390/genes15010099

**Published:** 2024-01-15

**Authors:** Jonathan B. Wagner, Susan Abdel-Rahman, Geetha Raghuveer, Andrea Gaedigk, Erin C. Boone, Roger Gaedigk, Vincent S. Staggs, Gregory A. Reed, Na Zhang, J. Steven Leeder

**Affiliations:** 1 Ward Family Heart Center, Children’s Mercy, Kansas City, MO 64108, USA; 2 Division of Clinical Pharmacology and Toxicology, Children’s Mercy, Kansas City, MO 64108, USA; 3 Department of Pediatrics, University of Missouri-Kansas City School of Medicine, Kansas City, MO 64108, USA; 4 Health Services & Outcomes Research, Children’s Mercy, Kansas City, MO 64108, USA; 5 Clinical Pharmacology Shared Resource, University of Kansas Cancer Center, Fairway, KS 66205, USA

**Keywords:** cholesterol, lipids, pediatrics, pharmacogenomics, pharmacokinetics, statins

## Abstract

This clinical study examined the influence of *SLCO1B1* c.521T>C (rs4149056) on plasma atorvastatin concentrations in pediatric hypercholesterolemia. The participants (8–21 years), including heterozygous (c.521T/C, n = 13), homozygous (c.521C/C, n = 2) and controls (c.521T/T, n = 13), completed a single-oral-dose pharmacokinetic study. Similar to in adults, the atorvastatin (AVA) area-under-concentration-time curve from 0 to 24 h (AUC_0–24_) was 1.7-fold and 2.8-fold higher in participants with c.521T/C and c.521C/C compared to the c.521T/T participants, respectively. The inter-individual variability in AVA exposure within these genotype groups ranged from 2.3 to 4.8-fold, indicating that additional factors contribute to the inter-individual variability in the AVA dose–exposure relationship. A multivariate model reinforced the *SLCO1B1* c.521T>C variant as the central factor contributing to AVA systemic exposure in this pediatric cohort, accounting for ~65% of the variability in AVA AUC_0–24_. Furthermore, lower AVA lactone concentrations in participants with increased body mass index contributed to higher exposure within the c.521T/T and c.521T/C genotype groups. Collectively, these factors contributing to higher systemic exposure could increase the risk of toxicity and should be accounted for when individualizing the dosing of atorvastatin in eligible pediatric patients.

## 1. Introduction

**Figure 1:**
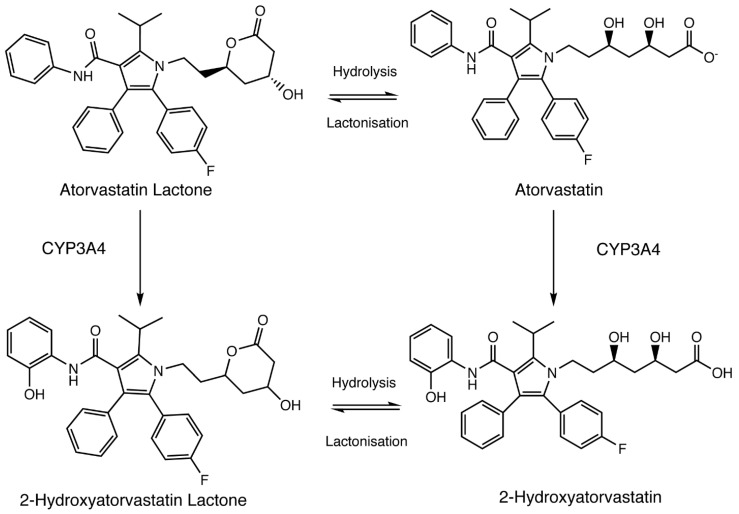
Atorvastatin pathway. Atorvastatin and atorvastatin lactone undergo CYP3A4-mediated biotransformation to form their respective 2-hydroxy metabolites.

Atorvastatin (AVA) is a moderately lipophilic, synthetic inhibitor of 3-hydroxy-3-methyl-glutaryl coenzyme A (HMG-CoA) reductase, labeled to treat children 10 years of age and older with heterozygous familial hypercholesterolemia [1,2]. AVA is given in its active acid form [3] and experiences hepatic uptake via the OATP1B1 and OATP1B3 proteins [4]. Cytochrome P450 (CYP)-mediated metabolism (e.g., CYP3A4) leads to the formation of an active metabolite, 2-hydroxyatorvastatin (Figure 1) [5,6]. AVA undergoes phase II metabolism (e.g., UGT1A1/3) to form inactive metabolites, atorvastatin lactone (AVL) and 2-hydroxyatorvastatin lactone (2-OH AVL) [7,8]. Following biotransformation, AVA is a substrate for MDR1 (e.g., P-Gp) and BCRP, which are responsible for hepatic clearance into the bile [9,10]. Given its lipophilic nature, AVA, similar to simvastatin acid, may experience more passive movement across extra-hepatic tissues (e.g., skeletal muscle, brain) compared to pravastatin or rosuvastatin [11,12,13,14,15,16,17]. However, a comparative study between lipophilic and hydrophilic statins and their differential effects on extra-hepatic cellular translocation leading to excessive systemic exposure and subsequent toxicity remains unknown. 

An approximate 40–60% decline in low-density lipoprotein cholesterol (LDL-C) has been demonstrated amongst three prospective AVA trials in children (ages 6–21 years), demonstrating its clinical effectiveness in the pediatric population [18,19,20]. Despite its effectiveness on a population basis, considerable (~2–5 fold) inter-individual variability in LDL-C has been observed. Of concern, in the only placebo-controlled trial on a pediatric cohort, nearly half of the participants did not reach target LDL-C levels (<130 mg/dL), even with self-reported adherence. Given the vast degree of variability in LDL-C decline and the dearth of longitudinal data on chronic statin exposure in a pediatric cohort, it is necessary that optimal statin dosing is established to maximize its effectiveness while minimizing its toxicity in a developing child.

To evaluate the inter-individual variability in LDL-C decline with statins, investigators must evaluate if poor or altered LDL-C decline is due to inadequate plasma AVA concentrations at the drug target (e.g., hepatocyte) or caused by inadequate drug target engagement due to the diminished expression and/or function of the targets of drug action. With respect to the former, hepatic uptake to the site of action within the liver represents the largest contributor to altered plasma statin concentrations. OATP1B1, encoded by the *SLCO1B1* gene, is a hepatic uptake protein and a major contributor to AVA hepatocellular transport [21,22]. The *SLCO1B1* variant NM_006446.5:c.521T>C (rs4149056) leads to an amino acid change (NP_006437.3:p.V174A), which leads to impaired OATP1B1 localization on the basolateral membrane [21]. As a result, this amino acid change in the OATP1B1 protein leads to decreased liver uptake and increased plasma AVA concentrations [23,24,25]. Previously, adult participants with *SLCO1B1* c.521T/C or c.521C/C genotypes had a ~1.5- to 2.5-fold increase in plasma AVA concentrations relative to those with the reference genotype (c.521T/T) [23]. The c.521T>C variant is relatively frequent (its global frequency is ~12% according to the gnomAD database; accessed 27 September 2023) and, according to the Pharmacogene Variation (PharmVar) Consortium [26,27], can occur in several haplotypes or star alleles, i.e., SLCO1B1*5, *15, *40, *46 and *47. Of these, four are designated by the Clinical Pharmacogenetics Consortium (CPIC) to have “no function”, while one (SLCO1B1*40) is of an “uncertain function”. 

However, the extrapolation of the adult c.521T>C genotype–phenotype relationship to a hypercholesterolemic child neglects the potential developmental differences in the expression of genes responsible for statin disposition. For example, we have recently demonstrated in a pediatric cohort that the influence of c.521T>C on simvastatin acid plasma concentrations was two-fold greater in children compared to adults, suggesting that this variant may be more impactful for a pediatric cohort [28]. In contrast, the genotype effect on a similar pediatric cohort receiving pravastatin and rosuvastatin was akin to the adult experience [23,29,30,31]. Surprisingly, in the aforementioned pediatric cohorts administered simvastatin and pravastatin, a significant within-c.521T>C-genotype variability in plasma statin concentration (~8- to 17-fold) was observed, with nongenetic factors partially contributing to the observed large range of plasma statin concentrations [28,29]. However, the magnitude of genetic and nongenetic factors is statin-dependent. Currently, the influence of pharmacogenomic and nonpharmacogenetic (i.e., developmental) factors on plasma AVA concentrations is not well established. 

Understanding the impact of drug disposition development (e.g., ontogeny) and c.521T>C on statin disposition is crucial to optimizing drug and dose recommendations for a pediatric cohort. Thus, the primary goal of this investigation was to establish the role of the *SLCO1B1* c.521T>C variant on plasma AVA concentrations in pediatric hypercholesterolemia. 

## 2. Methods 

### 2.1. Subjects

Those recruited from the Children’s Mercy Hospital Cardiology Pharmacogenomics Repository (CPR), a living biorepository and patient registry designed to facilitate genotype-stratified clinical trial participant selection, were screened for inclusion and exclusion criteria as previously reported [31]. Reference genotype (c.521T/T) and heterozygous variant populations (c.521T/C) were matched based on age, ethnicity and sex. Two additional participants with the c.521C/C genotype were enrolled as co-variates and not matched to the aforementioned groups. The study protocol was reviewed and approved by the Children’s Mercy Hospital Institutional Review Board, and the study was conducted in accordance with the US standards of Good Clinical Practice (ICH guidance E6(R3)). Informed permission/assent or consent was obtained from all those participating in the study.

### 2.2. Genetic Analysis

Genomic DNA was isolated from whole blood using a GeneElute^TM^ Mammalian Genomic DNA Miniprep Kit (Sigma, St. Louis, MO, USA) or a QIAamp^®^ DNA Blood Mini Kit (Qiagen, Valencia, CA, USA) according to the manufacturer’s protocols. DNA samples were genotyped for *SLCO1B1* c.-11187G>A (rs4149015), c.388A>G (p.N130D, rs2306283) and c.521T>C (p.V174A, rs4149056) using commercially available TaqMan^®^ single-nucleotide polymorphism (SNP) genotyping tests (Thermo Fisher Scientific, Waltham, MA, USA) with KAPA Probe Fast qPCR Master Mix (2X) ABI Prism^®^ (KAPA Biosystems, Boston, MA, USA) on a QuantStudio12k Flex Real-Time PCR System (Thermo Fisher Scientific, Waltham, MA, USA). Cycling conditions for all assays were as recommended by the manufacturer. Coriell Institute for Medical Research (Camden, NJ, USA) DNA samples were used as controls. Twenty percent of the samples were chosen and reanalyzed for quality control and were concordant with the initial data. 

The samples were subjected to a targeted next-generation sequencing (NGS) panel *a posteriori* to test additional variants in *SLCO1B1* and variants of other genes that influence AVA disposition [23,32,33,34,35,36], as noted above in the first introductory paragraph. These included the following *genes* (proteins) of interest in AVA disposition: *SLCO1B3* (OATP1B3), *ABCB1* (MDR1 or P-gp), *ABCG2* (BCRP)*, CYP3A4* (CYP3A4)*, CYP3A5* (CYP3A5), *UGT1A1* (UGT1A1) and *UGT1A3* (UGT1A3). In short, a TruSeq Library constructed according to the manufacturer’s protocol (Illumina, San Diego, CA, USA) and a custom targeted capture sequencing panel, ADME Panel (Integrated DNA Technologies, Coralville, IA, USA), were used for data enhancement [37]. Samples were sequenced on a MiSeq instrument (Illumina, San Diego, CA, USA) with paired-end 200-base pair reads. The read coverage was approximately 300×. Variants within the gene regions of interest were retrieved with NGS using bcftools (v1.7-8). For genes with a star allele nomenclature, calls were made manually for *SLCO1B1*, *CYP3A4* and *CYP3A5* based on PharmVar allele definitions and for *UGT1A1* and *UGT1A3*, using the *UGT* Nomenclature site at https://www.pharmacogenomics.pha.ulaval.ca/ugt-alleles-nomenclature/ (accessed on 8 December 2023).

### 2.3. Study Design

This single-center, open-label, genotype-stratified, single-oral-dose pharmacokinetic study compared plasma AVA concentrations amongst pediatric hypercholesterolemic patients with one or two *SLCO1B1* variant alleles (c.521T/C and T/T) to patients who had the reference genotype (c.521T/T). Those on statin therapy required a 7-day washout prior to the study visit. A screening physical examination (including sexual maturity rating via Tanner Staging) was performed prior to ingesting AVA. 

A single oral dose of AVA (ages 8–21 years: 10 mg tablet, Greenstone Pharmaceuticals^®^, Lot Number L57138) was given with water after an overnight fast, and no meals were allowed for 3 h after AVA ingestion. Venous blood samples were drawn from an intravenous line prior to AVA ingestion (time 0) and at 0.5, 1, 1.5, 2, 2.5, 3, 4, 5, 7, 9, 18 and 24 h post-ingestion to measure plasma AVA and AVA metabolite concentrations. Samples were immediately centrifuged at 4 °C for 10 min at 600 g. Plasma was removed and stored at −80 °C until the analysis. 

### 2.4. Analytical Methods 

Human plasma (50 µL) containing AVA, 2-hydroxy atorvastatin (2-OH AVA), atorvastatin lactone (AVL) and 2-hydroxy atorvastatin lactone (2-OH AVL) was spiked with 200 µL of cold 0.1% acetic acid in acetonitrile containing the combined internal standards (IS) (10 ng/mL) atorvastatin-d5, 2-hydroxy atorvastatin-d5, atorvastatin-d5 lactone and 2-hydroxy atorvastatin lactone-d5. All analytical reference compounds were purchased from Toronto Research Chemicals at their highest purity. Plasma proteins were precipitated with acetonitrile and centrifuged, and the filtrates were transferred to a new set of clean, labeled microcentrifuge tubes. An aliquot of the extracts was then transferred to a 96-well plate and analyzed by ultra-performance liquid chromatography tandem mass spectrometry (UPLC-MS/MS) with positive-ion electrospray ionization using multiple-reaction monitoring (MRM). 

A Waters ACQUITY UPLC HSS C18 (3.5 μm, 2.1 × 100 mm) separated the analytes. Gradient elution was optimized with an acetonitrile–water–MeOH gradient: mobile phase A: water: MeOH: acetic acid (*v*/*v*/*v*), 90:10:0.1; mobile phase B: acetonitrile: methanol: acetic acid (*v*/*v*/*v*), 60:40:0.1. The yielded retention times were 2.54 min for AVA, 2.18 min for 2-OH AVA, 2.85 min for AVL and 2.45 min for 2-OH AVL. For the analytes, the MRM transitions 559.29 > 440.12 (AVA), 541.29 > 448.14 (AVL), 575.29 > 440.14 (2-OH AVA) and 557.27 > 448.11 (2-OH AVL) were used for quantitation. And for the IS, the MRM transitions 564.32 > 445.16 (AVA-d5), 546.31 > 453.15 (AVL-d5), 580.32 > 445.17 (2-OH AVA-d5) and 562.30 > 453.15 (2-OH AVL-d5) were used for quantification. All the data acquisition and analysis were carried out by Masslynx 4.2 software.

The validated linear range for the quantification of all the analytes was 0.5–100 ng/mL; the LLOQ (lower limit of quantification) for this assay is 0.5 ng/mL for each analyte. The linearity for all the analytes occurred in the range of 0.5–100 ng/mL. Intra- and inter-day precisions of ≤15% were used for three quality control levels (LQC: 1 ng/mL, MQC: 5 ng/mL, HQC: 50 ng/mL) and ≤20% for the LLOQ. The accuracy of the QC samples ranged from 80% to 120% of the theoretical concentrations at the LLOQ level and 85% to 115% at the three other quality control levels for atorvastatin and three metabolites. 

### 2.5. Pharmacokinetic Parameters

Kinetica version 5.0 (Thermo Fisher Scientific, Philadelphia, PA, USA) was utilized for all plasma AVA analyses to generate the necessary pharmacokinetic parameters. Plasma AVA concentration vs. hours post dosing data for AVA, 2-OH AVA, AVL and 2-OH-AVL were curve fitted using a peeling algorithm to generate initial monoexponential parameter estimates. The final estimates of the terminal elimination rate constant (λ_z_) were determined from a similar algorithm used previously [28,29,31]. The individual peak plasma concentration (Cmax) and time to maximal concentration (Tmax) were obtained by direct examination of the plasma AVA concentration versus hour profile. The area under the plasma concentration versus time curve during the sampling period (AUC_0–n_) was calculated using the mixed log–linear method, where n refers to the final sampling time with quantifiable plasma AVA or AVA metabolite concentrations. To determine if the AVA metabolites were driven by an elimination rate-limited vs. formation rate-limited process, a compartmental pharmacokinetic approach was performed on 23 participants with sufficient time points to accurately determine both the formation and elimination rate constants. 

### 2.6. Statistical Analyses

Pharmacokinetic parameters were examined in JMP^®^ version 14 (SAS, Cary, NC, USA). Pharmacokinetic parameters reflective of plasma AVA concentrations (Cmax, AUC_0–n_) and the *SLCO1B1* genotype (c.521T/T vs. T/C and C/C) were dependent variables and independent variables, respectively. The Kruskal–Wallis test was leveraged to compare the pharmacokinetic parameters related to genotype groups and demographics. 

Linear regression to examine the association between the AVA AUCn and c.521T>C genotype groups, adjusting for BMI *z*-score, sex and Tanner score, was performed. For modeling, the genotype was captured by coding the number of c.521C alleles (0, 1, or 2) and treating this as a quantitative variable. A single Tanner score was computed by averaging each participant’s Tanner breast/testicular and Tanner public hair stage scores. AVA AUC_0–24_ was first modeled as a function of BMI *z*-score, sex and Tanner score to estimate the proportion of variability that could be accounted for by these participant characteristics. Then, the count of c.521C alleles was added to the model to estimate the additional variability accounted for.

AVA given at a fixed dose resulted in an almost 4-fold range of weight-based doses (0.07–0.28 mg/kg). Therefore, the Cmax and AUC were normalized to a dose for each individual participant by dividing the exposure parameter value by the actual mg/kg dose received, then multiplying by the mean mg/kg dose for the entire cohort (e.g., participant AUC (ng·hr/mL)/participant dose (mg/kg) × cohort mean dose (mg/kg)).

## 3. Results

### 3.1. Participant Demographics and Adverse Events

Twenty-eight participants (fifteen males, thirteen females) successfully completed the study, and no participants withdrew from the study. The participant demographics were similar between the *SLCO1B1* c.521 T/C groups (Table 1). This includes similar mg/kg doses received between the genotype groups. No adverse events were reported during the study.

### 3.2. Drug Disposition Profiles

**Figure 2:**
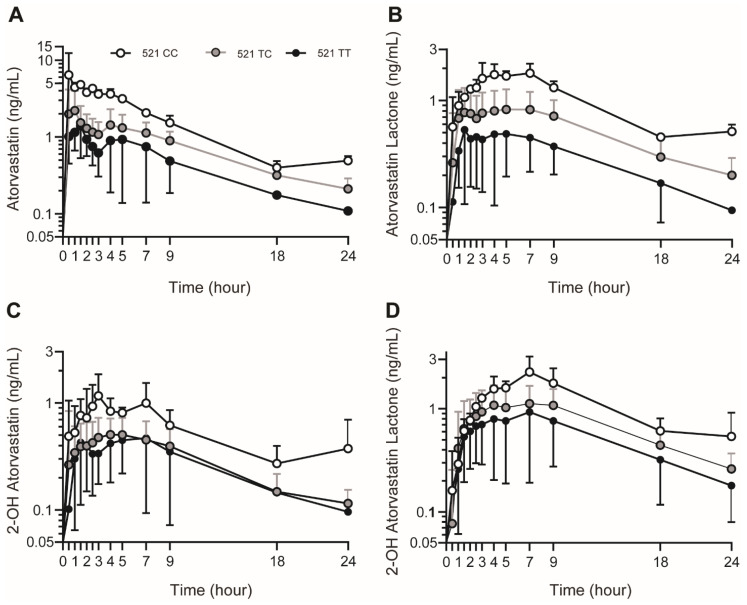
(**A**) Mean ± SD plasma analyte concentrations (ng/mL) of atorvastatin, (**B**) atorvastatin lactone, (**C**) 2-hydroxyatorvastatin, and (**D**) 2-hydroxyatorvastatin lactone. Black, gray and open white circles denote participants with the c.521T/T (n = 13), c.521T/C (n = 13) and c.521C/C (n = 2) genotypes, respectively.

The drug disposition profiles of plasma AVA, AVL, 2-OH AVA and 2-OH AVL (Figure 2A–D) reflected first-order absorption and elimination. Plasma AVA, AVL, 2-OH AVA and 2-OH AVL concentrations were present in all the participants. Secondary AVA peaking, suggestive of entero-hepatic recirculation, in 12 participants (43%) precluded an accurate assessment of the mean terminal elimination rate constant across the population. 

A relatively large plasma AVA concentration range (~nine-fold) was found (median (IQR), Cmax: 2.2 ng/mL (1.6–3.5 ng/mL); and AUC_0–24_: 17.1 ng·hr/mL (11.5–19.7), Table S1). Significant variability was also observed for AVL (median (IQR), Cmax: 0.9 ng/mL (0.7–1.2); AUC_0–24_: 8.7 ng·hr/mL (4.0–15.0), Table S1), 2-OH AVA (median (IQR) Cmax: 0.7 ng/mL (0.5–1.0 ng/mL); AUC_0–24_: 6.4 ng·hr/mL (4.8–9.4), Table S1) and 2-OH AVL (median (IQR) Cmax: 1.1 ng/mL (0.8–1.6 ng/mL); AUC_0–24_: 14.5 ng·hr/mL (5.5–24.1), Table S1).

### 3.3. SLCO1B1 c.521T>C Genotype Influence on Plasma AVA and AVA Metabolite Concentrations

**Figure 3:**
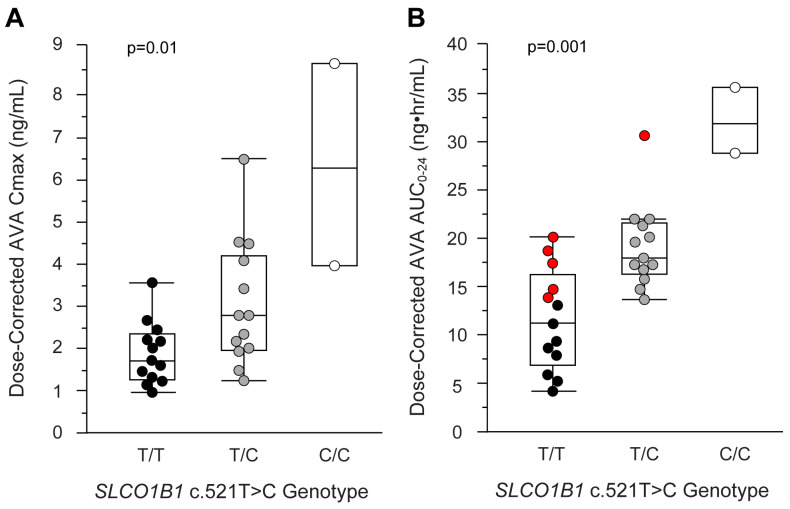
(**A**) Relationship of atorvastatin C_max_ (ng/mL) and (**B**) AUC_0–24_ (ng·hr/mL) normalized for doses amongst the *SLCO1B1* c.521 genotype groups. Black, gray and open white circles denote participants with the c.521T/T (n = 13), c.521T/C (n = 13) and c.521C/C (n = 2) genotypes, respectively. Red circles denote “high-exposure subgroup” participants (c.521T/T, n = 5; c.521T/C, n = 1).

The maximum mean AVA concentrations (Cmax) were 3.6-fold higher in the c.521C/C (n = 2) group and 2.8-fold higher in the c.521T/C (n = 13) group relative to the c.521T/T (n = 13) group (Figure 3A, Table 2). The mean AVA AUC_0–24_ was 2.8-fold higher in the c.521C/C group and 1.7-fold higher in the c.521T/C group relative to the participants with the reference genotype (Figure 3B, Table 2). 

The genotype also had an effect on the AVL Cmax and AUC_0–24_. The Cmax was 1.6-fold and 2.1-fold higher in the c.521T/C and C/C groups, respectively, relative to the c.521T/T group (Table 2). Those with the c.521T/C genotype had a 1.8-fold higher mean AVL AUC_0–24_ compared with the participants with the reference genotype (Table 2). 

2-OH AVA exposure was similar amongst the genotype groups (*p* = 0.14; Table 2). Similarly, 2-OH AVL exposure was also not affected by genotype (*p* = 0.19; Table 2).

The *SLCO1B1* c.-11187G>A or c.388A>G genotype demonstrated no correlation to the plasma AVA, AVL, or 2-OH AVA concentrations. There was a difference in the 2-OH AVL Cmax amongst the c.388A>G genotype groups (*p* = 0.04); however, those in the c.388G/G genotype group had a younger average age, which influenced this association.

Interestingly, variability in the plasma AVA concentrations within the genotype groups was also observed. The largest range of plasma AVA concentrations occurred in the c.521T/T group (4.8-fold) compared to the c.521T/C group (2.3-fold). These data indicate that additional patient-specific factors, in combination with their c.521T>C genotype, influence AVA systemic exposure. AVA exposure was normally distributed within the c.521T/T and c.521T/C groups (Figure S1). However, five c.521T/T participants had a high AVA exposure that fell within the range of that of the c.521T/C group, and one c.521T/C participant had a high AVA exposure that fell within the range of that of the c.521C/C group (Figure 3B). These participants were identified as a “high-exposure subgroup”. Therefore, secondary-hypothesis-generating post hoc analyses were conducted to discover additional variables that could explain the large range of plasma AVA concentrations within the *SLCO1B1* c.521 genotype groups. 

### 3.4. Demographic and Anthropometric Influence on Plasma AVA and AVA Metabolite Concentrations

Setting a threshold for multiple testing of *p* = 0.0011 using the Bonferroni method (*p* = 0.05/44, or 0.0011), AUC_0–24_ for AVL and 2-OH AVL, but not AVA and 2-OH AVA, was associated with height, weight and body mass index (Table S2). The exposure metrics for all the analytes were generally comparable across sex, race and ethnicity groups (Table S3).

### 3.5. Non-SLCO1B1 Drug Transporter Influence on Plasma AVA and AVA Metabolite Concentrations

The gene sequencing of hepatic transporters associated with atorvastatin uptake (*SLCO1B1*, *SLCO1B3)* and efflux (*ABCB1*, *ABCG2*) was carried out (Table S4) and did not reveal any variants impacting the plasma AVA and AVA metabolite concentrations. Some of the variants on Table S4 could explain the generous plasma AVA concentrations for some of the participants in the high-exposure subgroup, but none were unique to this group.

### 3.6. Effect of CYP3A and UGT on Plasma AVA and AVA Metabolite Concentrations

Compartmental pharmacokinetic analyses were performed on a cohort of participants (n = 23) with sufficient time points to generate elimination rate half-lives for AVA, 2-OH AVA, AVL and 2-OH AVL, which demonstrate similar terminal slopes, suggesting a formation rate-limited process. Thus, metabolite ratios were justified for further analyses. 

To examine altered CYP-mediated metabolism as a possible contributor to the higher plasma AVA concentrations within the high-exposure subgroup, plasma 2-OH AVA, as a percentage of the total analyte (AVA + AVL + 2-OH AVA + 2-OH AVL), was quantitated. A low ratio could suggest that less 2-OH AVA formation occurred, resulting in the higher observed AVA systemic exposure. For the entire cohort, a linear regression model comparing AVA AUC_0–24_ and 2-OH AVA to the total analyte ratio was applied, and no correlation was observed. In the aforementioned “high-exposure subgroup”, a lower 2-OH AVA-to-total-percentage trend was not observed compared to others in the same genotype group. One c.521T/T high-exposure-subgroup participant had one of the lowest 2-OH AVA-to-total-analyte percentages, which could explain their excessive plasma AVA concentration compared to the others in the same genotype group. Of note, that participant had a genotype associated with normal CYP3A4 activity (e.g., they had CYP3A4*1/*1) and was a nonexpressor of *CYP3A5* (e.g., they had CYP3A5*3/*3) (Table S4). Collectively, the *CYP3A4*/*5* genotypes were noninformative relative to characterizing those in the high-exposure subgroup.

To examine if the diminished lactonization in the “high-exposure subgroup” was associated with increased plasma AVA concentrations, plasma AVL, as a percentage of the total analyte (AVA + AVL + 2-OH AVA + 2-OH AVL), was quantitated. For the entire cohort, a linear regression model comparing AVA AUC_0–24_ and AVL-to-the-total analyte ratio was applied, and no correlation was observed. Four “high-exposure-subgroup” participants (one *SLCO1B1* c.521/TC and three c.521T/T) were within the lower half of the AVL-to-the-total percentage normal quantile distribution plot for each c.521T>C genotype group. One of these four participants with a low AVL-to-total-analyte percentage had sequence variants associated with diminished UGT1A3 function (e.g., *UGT1A3* *1/*8) (Table S4). 

**Figure 4:**
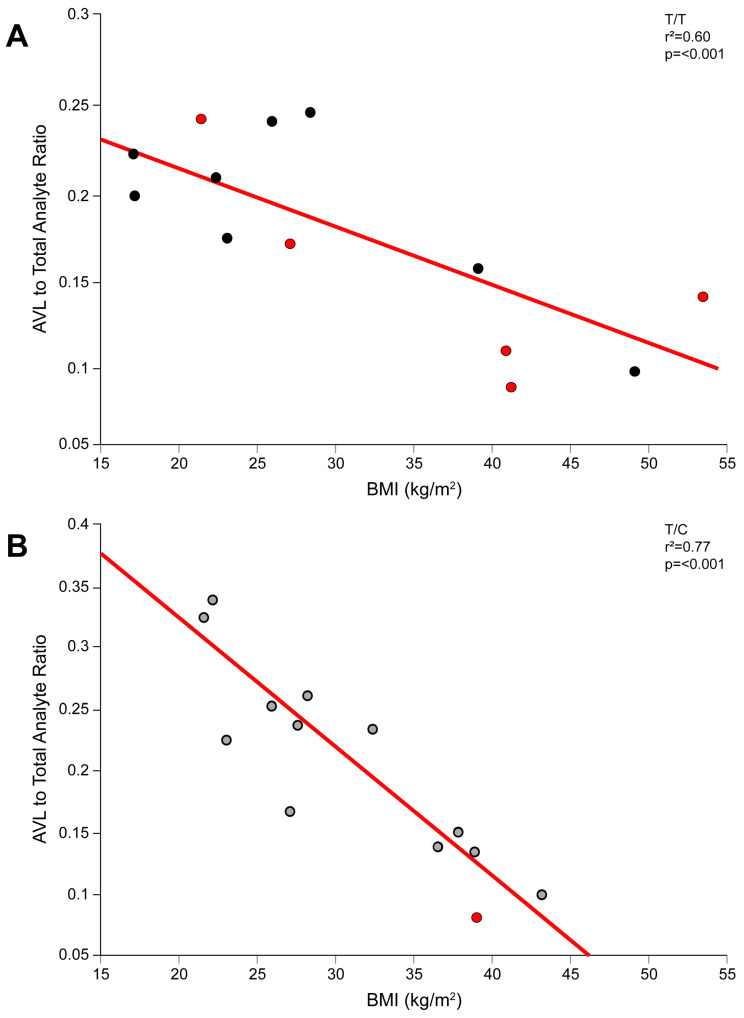
Correlation between AVL + 2-OH AVL-to-total-analyte ratio and BMI in the (**A**) c.521T/T and (**B**) *SLCO1B1* c.521T/C genotype groups. Black and gray circles denote participants with the c.521T/T (n = 13) and c.521T/C (n = 13) genotypes, respectively. Red circles denote “high-exposure-subgroup” participants (c.521T/T, n = 5; c.521T/C, n = 1).

Given the observation of a negative correlation between AVL and BMI, as previously mentioned in Table S2, we evaluated the AVL-to-total-analyte percentage based on the genotype groups. Interestingly, a significant correlation was observed between the AVL-to-total-analyte percentage and BMI amongst both genotype groups (Figure 4). Four of the “high exposure subgroup” were among the highest BMIs for their respective genotype cohorts. Collectively, enhanced adiposity could be a factor correlated with impaired lactonization and subsequently contribute to the variability within the c.521T>C genotype groups.

### 3.7. Plasma AVA Concentration Multivariate Model

In the multivariate modeling of AVA AUC_0–24_, the model, including the BMI *z*-score, sex and the Tanner score, yielded an R-squared value of 0.09. The addition of the *SLC01B1* genotype (coded as the number of c.521C alleles) to the model increased the R^2^ to 0.65, meaning 65% of variability in AVA AUC_0–24_ could be accounted for by the four explanatory variables (Table 3). According to the model estimates, a difference of one c.521C allele is associated with a difference of 9.0 ng·hr/mL (equivalent to 1.2 SDs) in AVA AUC_0–24_, adjusting for patient BMI *z*-score, sex and tanner score, with a 95% CI of 6.0–12.0 (*p* < 0.001). 

## 4. Discussion

This study examined the influence of the *SLCO1B1* c.521T>C variant on the plasma AVA concentration in a pediatric cohort. The magnitude of the effect for the c.521T/C group compared to the reference c.521T/T group in the pediatric cohort was analogous to that reported in adults (i.e., 1.5-fold, as observed by Pasanen et al. [23], vs. 1.7-fold in this study). Additionally, a difference was observed between the c.521T/T reference and c.521C/C groups, which is also consistent with the aforementioned study in adults. We observed a difference in AVA systemic exposure amongst the three c.521T>C genotype groups. This is consistent with our previous analyses involving simvastatin acid, pravastatin and rosuvastatin, where systemic exposure increased by approximately two-fold, on average, with each variant “C” allele present [28,29,31]. In adults, a statistically significant difference amongst each genotype group has only been observed with atorvastatin [23,30,38]. The magnitude of the *SLCO1B1* c.521T>C effect on plasma AVA concentrations is comparable to that found for simvastatin acid and more influential than that observed with rosuvastatin and pravastatin in this pediatric cohort [28,29,31]. 

Consistent with our previous investigations in children, we observed extensive and potentially clinically meaningful variability in the AVA systemic exposures within the c.521T/T (4.8-fold) and c.521T/C (2.3-fold) groups (TT: 4.1–19.7 ng·hr/mL; TC: 13.5–30.9 ng·hr/mL; Figure 3B). This has been described in adults, with coefficients of variation for the AVA AUC ranging from 31 to 57% within these genotype groups [23]. Similar to our experience with pravastatin, simvastatin and rosuvastatin, these data indicate that the *SLCO1B1* c.521 genotype, in combination with other patient-specific factors, influences AVA exposure, and these factors need to be accounted for when optimizing atorvastatin treatment in an individual child or adolescent. 

OATP1B1, OATP1B3, MDR1(P-gp) and BCRP are proteins known to transport AVA [4,9,10]. Gene sequencing was completed later on in the study to determine if genetic factors unique to the “high-exposure subgroup” existed (Table S4). For the *SLCO1B1* phenotype, the next-generation sequencing data were all consistent with the functional status (e.g., c.521T/T: normal, c.521T/C: decreased) [26]. In our previous investigation on the same cohort of children and adolescents taking rosuvastatin [31], three “high-exposure-subgroup” participants (one c.521T/T and two c.521T/C) had nonsynonymous variants of *SLCO1B3* (c.767G>C, p.G256A). In this analysis with atorvastatin, only one high exposure subgroup participant (c.521T/C) had a *SLCO1B3* c.767G/C genotype. *SLCO1B3* c.767G/C has been reported to be associated with a diminished expression of OATP1B3. However, from a functional standpoint, this has not been associated with diminished cellular transport of probe substrates [39]. However, it is appreciated that variants in hepatic uptake transporters can be substrate-specific [40]. The *SLCO1B3* c.767G>C genotype’s influence on the rosuvastatin or atorvastatin distribution has not been investigated but should be assessed in future cellular transport analyses. 

The *ABCB1* (MDR1/P-Gp) and *ABCG2* (BCRP) genes encode enterocyte and hepatocyte efflux transporters and are proteins associated with the transcellular movement of AVA into the intestinal lumen or bile canaliculus [41]. Therefore, sequence variation resulting in diminished protein expression can result in enhanced absorption, leading to higher systemic exposure. In fact, Keskitalo *et al*. have demonstrated that subjects with *ABCB1* genotypes had higher plasma statin concentrations relative to the reference genotypes [32]. Four of the high outliers described by Keskitalo *et al*. had one variant in *ABCB1* (c.3435T>C, p.I1145=; c.2677G>T/A, p.S893A/T; or c.1236T>C, p.G412=). However, in our investigation, nobody in the “high-exposure subgroup” had *ABCB1* c.3435T/T, c.2677T/T or c.1236T/T associated with a significantly higher AVA exposure. Two *ABCG2* variants (c.34G>A and c.421C>A) associated with increased atorvastatin systemic exposure in adults [33] were evaluated in our cohort. Two out of the six participants with *ABCG2* c.34G>A SNP were in the “high-exposure subgroup”. Collectively, these variants in genes involved in AVA transport were not unique to the “high-exposure subgroup” and therefore are unlikely to explain the high AVA exposure on their own within the *SLCO1B1* c.521 genotype groups or may only have a small effect. 

The contribution of interindividual *CYP3A* variation was evaluated in our study to determine whether the higher AVA systemic exposure could be explained by impaired AVA metabolism. There was one “high-exposure-subgroup” participant with the lowest 2-OH AVA-to-total-analyte percentage within the c.521T/T group, suggesting that a low biotransformation from AVA to 2-OH AVA results in higher AVA systemic exposure. However, no variants were identified in this individual that would explain the altered CYP3A4/5 activity relative to the rest of the individuals within the c.521T/T group. Collectively, the discordant biotransformation of AVA may have contributed to the excessive AVA exposure in that one participant, but it does not explain the higher AVA exposure for the remaining five “high-exposure-subgroup” participants. 

The contribution of variable lactonization to excessive AVA systemic exposure was also evaluated. If AVA lactonization was impaired, it could lead to a “high-exposure subgroup” within the c.521 genotype groups. Amongst the six high-exposure-subgroup participants, four had a significantly lower lactone-to-total-analyte percentage. However, only one “high-exposure-subgroup” participant had sequence variants that are known to diminish UGT1A3 activity (Table S4) [36]. Thus, variation in *UGT1A* may contribute to the discordant results only in one “high-exposure-subgroup” participant, but it does not explain the higher AVA exposure for the remaining four members of the high-exposure subgroup.

Previously, we demonstrated that BMI, in conjunction with genetic variation in *SLCO1B1*, is linked with excessive plasma pravastatin concentrations [29]. Obesity is associated with the development of nonalcoholic fatty liver disease (NAFLD) in pediatric and adults cohorts [42,43]. NAFLD contributes to variable hepatic uptake transporter expression [44,45,46], leading to altered simvastatin and pravastatin transport [46,47]. We did not observe a correlation between BMI and AVA systemic exposure (Table S2). However, a negative correlation between plasma AVL and 2-OH AVL concentrations and BMI was noted. Perhaps more striking was the observation of a significant negative correlation between the AVL-to-total-analyte percentage and BMI (Figure 4), potentially contributing to the altered AVA exposure in the “high-exposure subgroup”. Diminished lactonization in the obese population has previously not been described. In fact, to the contrary, Xu et al. demonstrated in a murine model that UGT expression was increased in obese mice compared to controls [48]. In human livers, there was no difference with regards to *UGT1A3* mRNA expression in liver tissue samples with liver adiposity and fibrosis compared to controls [49]. However, the role of liver adiposity on drug disposition (e.g., drug transport and lactonization) for children and adolescents remains unknown and requires further elucidation. 

Another explanation for the variable plasma AVA concentrations lies in the degree of transformation of AVA to AVL in the acidic conditions of the stomach after drug ingestion. Kearney et al. demonstrated that at a pH < 6, an equilibrium between AVA and AVL exists but favors AVA. At a more basic pH > 6, the equilibrium is no longer detected and greatly favors AVA [50]. More recently, atorvastatin physiologically based pharmacokinetic (PBPK) modeling, including in vitro gastric acid–lactone conversion alterations, successfully reproduced observed AVA PKs. Additionally, it was shown that delayed gastric emptying explains the increased conversion of AVA to AVL. The limited data amongst the pediatric obese patient population demonstrate that the gastric emptying time is delayed with increasing abdominal obesity, suggesting that an increased conversion to AVL should have occurred [51]. Further delays in the emptying time in pediatric obese patients with symptomatic gastroesophageal reflux (GERD) events compared to obese children without GERD can occur. In our study cohort, two of the six “high-exposure-subgroup” participants had a history of GERD. Those participants had previously undergone acid suppression therapy (omeprazole, ranitidine), which was held two weeks prior to the study enrollment. In adults, the gastric emptying time is usually shorter in obese patients compared to nonobese controls [52,53,54]. Given the age of the cohort, our obese cohort could be more analogous to adult parameters in terms of gastric emptying time, resulting in diminished lactonization at the level of the stomach. Taken together, the etiology of lower AVL exposure in those participants with higher BMIs requires further study, as we were not adequately powered to elucidate such an effect. 

## 5. Conclusions

Our study establishes that the *SLCO1B1* c.521 genotype influences the plasma AVA concentration variation observed in children and adolescents dosed with atorvastatin. However, as we have observed with our previous studies involving pravastatin, simvastatin and rosuvastatin [28,29,31], within each genotype group, there remains a degree of variation that the *SLCO1B1* c.521 genotype alone or other variants within this gene can explain. As was observed with rosuvastatin, no additional genetic variants associated with protein aberrations involved in atorvastatin disposition contributed to those in the high-exposure subgroup. The effect of AVA lactonization, especially on the obese child, requires further exploration as it may be an additional variable influencing AVA systemic exposure. Given that atorvastatin lactone is not active, altered lactonization could potentially lead to an altered atorvastatin response (e.g., LDL-C reduction). This could be further altered in the obese population that is prescribed atorvastatin. Similar to rosuvastatin, atorvastatin had a lower variation in plasma AVA concentration for individuals within the genotype groups compared to pravastatin and simvastatin [28,29]. Given the lower variation in plasma AVA concentrations, rosuvastatin and/or atorvastatin may be preferred for children with hypercholesterolemia prescribed a statin. However, determining the impact of atorvastatin or rosuvastatin on the biomarkers of long-term LDL-C decline requires further analysis before one can conclude that they are preferred agents in the treatment of pediatric hypercholesterolemia. 

## Figures and Tables

**Table 1:** Participants stratified by *SLCO1B1* c.521T>C genotype.

	*SLCO1B1* c.521T/T (n = 13)	*SLCO1B1* c.521T/C (n = 13)	*SLCO1B1* c.521C/C (n = 2)
Age (years)	15.1 (±3.0)	14.9 (±2.9)	14.3 (±4.3)
Weight (kg)	85.7 (±34.5)	87.0 (±26.0)	62.2 (±1.6)
Height (cm)	164.1 (±8.5)	165.3 (±11.8)	157.6 (±19.8)
BMI (kg/m^2^)	31.6 (±12.0)	31.2 (±6.9)	25.6 (±5.7)
Gender			
Female	6	6	1
Male	7	7	1
Ethnicity			
White, nonHispanic	7	7	1
White, Hispanic	5	5	1
African-American	1	1	0
Tanner Breast/Testicular			
Stage 1	0	0	0
Stage 2	2	2	1
Stage 3	0	1	0
Stage 4	4	0	0
Stage 5	7	10	1
Pubic			
Stage 1	1	0	0
Stage 2	0	3	1
Stage 3	1	0	0
Stage 4	3	0	0
Stage 5	8	10	1
Dose (mg/kg)	0.14 (±0.06)	0.13 (±0.04)	0.16 (±0.00)

All data expressed as mean (±SD).

**Table 2:** Atorvastatin and atorvastatin analyte parameters stratified by *SLCO1B1* c.521T>C genotype.

	*SLCO1B1* c.521T/T (n = 13)	*SLCO1B1* c.521T/C (n = 13)	*SLCO1B1* c.521C/C (n = 2)	*p*
**Atorvastatin**				
C_max_ (ng/mL)	1.8 (±0.7)	3.0 (±1.5)	6.4 (±3.3)	0.01
t_max_ (h)	1.0 (0.5–5.0)	1.0 (0.5–7.0)	1.0 (0.5–1.5)	N/A
AUC_0–24_ (ng·hr/mL)	11.3 (±5.1)	19.1 (±4.5)	32.0 (±5.2)	0.001
**Atorvastatin** **Lactone**				
C_max_ (ng/mL)	0.7 (±0.4)	1.1 (±0.3)	1.5 (±0.4)	0.007
t_max_ (h)	2.5 (1.0–9.2)	4.0 (1.0–9.0)	6.0 (5.0–7.0)	N/A
AUC_0–24_ (ng·hr/mL)	7.2 (±4.8)	13.0 (±10.1)	27.8 (±2.3)	0.03
**2-Hydroxy Atorvastatin**				
C_max_ (ng/mL)	0.7 (±0.2)	0.8 (±0.3)	1.3 (±0.1)	0.09
t_max_ (h)	2.0 (1.0–7.0)	3.0 (0.5–7.0)	5.0 (3.0–7.0)	N/A
AUC_0–24_ (ng·hr/mL)	6.2 (±2.8)	7.5 (±3.2)	10.8 (±2.2)	0.14
**2-Hydroxy Atorvastatin Lactone**				
C_max_ (ng/mL)	1.0 (±0.5)	1.3 (±0.4)	1.9 (±0.8)	0.11
t_max_ (h)	7.0 (1.5–9.2)	5.0 (2.0–7.0)	7.0 (7.0–7.0)	N/A
AUC_0–24_ (ng·hr/mL)	14.0 (±11.9)	17.4 (±12.7)	31.0 (±9.4)	0.19

Data expressed as mean (±SD); t_max_ expressed as median (range). Kruskal–Wallis test used for all analyses.

**Table 3:** Results of multivariate model for AVA AUC_0–24_.

Explanatory Variable	β (95% CI)	*p*
BMI *z*-score	1.4 (−0.4, 3.1)	0.114
Male sex	1.7 (−2.2, 5.6)	0.378
Tanner score	0.5 (−1.1, 2.2)	0.524
*SLCO1B1* c.521 number of “C” alleles	9.0 (6.0, 12.0)	<0.001

## Data Availability

Data are contained within the article and supplementary materials.

## Supplementary Materials

The following supporting information can be downloaded at: https://www.mdpi.com/article/10.3390/genes15010099/s1, Figure S1: The normal distribution plots for AVA AUC0-24 (ng·hr/mL) amongst the c.521T/C and c.521T/T genotypes. Gray circles denote participants with the c.521T/C genotype (n = 12). Black circles denote participants with the c.521T/T genotype (n = 7). Red circles denote “high exposure subgroup” participants (c.521T/T, n = 5; c.521T/C, n = 1).; Table S1: Pharmacokinetic parameters amongst the entire cohort; Table S2: Correlations between demographic parameters and systemic exposure; Table S3: Atorvastatin and atorvastatin analyte parameters stratified by demographic and developmental parameters; Table S4: Drug transporter and metabolism gene sequencing results.

## Abbreviations

2-OH-AVA—2-hydroxy atorvastatin; 2-OH-AVL—2-hydroxy atorvastatin lactone; ABC—ATP binding cassette; AUC—area under the curve; AVA—atorvastatin; AVL—atorvastatin lactone; BMI—body mass index; BCRP—Breast cancer resistance protein; Cmax—peak plasma concentration; CPIC—Clinical Pharmacogenetics Consortium; CPR—Cardiology Pharmacogenomics Repository; CYP—cytochrome P450; DNA—deoxyribonucleic acid; HMG-CoA—3-hydroxy-3-methyl-glutaryl coenzyme A; HQC—high quality control; IQR—interquartile range; LDL-C—low-density lipoprotein cholesterol; LLOQ—lower limit of quantification; LQC—lower quality control; MDR1—multidrug resistance protein; MQC—middle quality control; NGS--next-generation sequencing; OATP—organic anion transporting polypeptide; PBPK—physiologically based pharmacokinetic; P-gp—P-glycoprotein; SLC—solute carrier family gene; SNP—single-nucleotide polymorphism; t_max_—time to maximal concentration; UGT—UDP-glucuronosyltransferase.
