# Supplementary material for: SLCO1B1 Genetic Variation Influence on Atorvastatin Systemic Exposure in Pediatric Hypercholesterolemia
Source: Genes (Basel). 2024 Jan 15;15(1):99. doi: 10.3390/genes15010099 (PMC10815823; doi:10.3390/genes15010099)
Supplement: Supplementary file 1 [file genes-15-00099-s001.zip › genes-2797837-supplementary materials.pdf]

Supplementary Figures

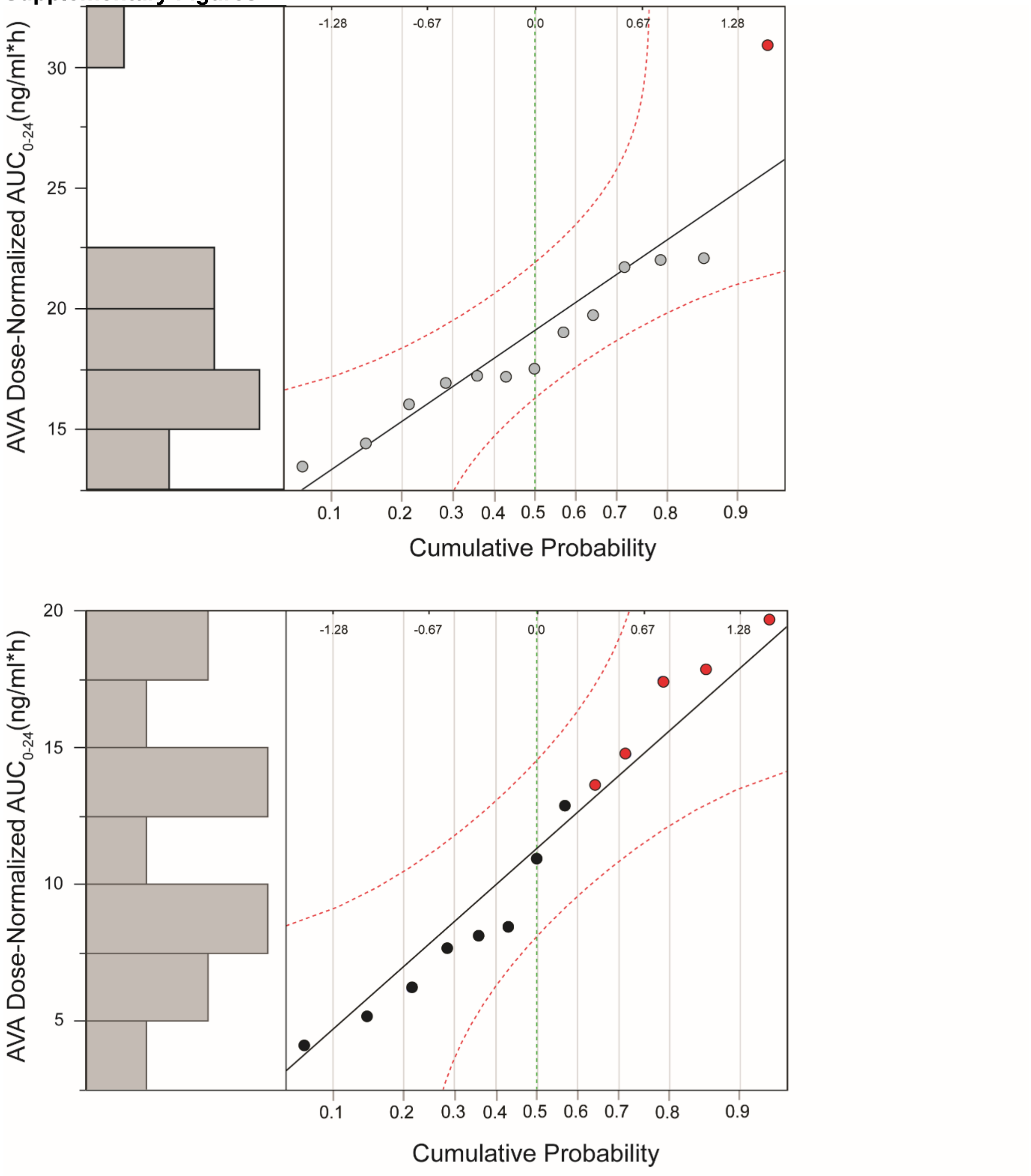

Supplemental Figure S1. The normal distribution plots for AVA AUC0-24 (ng/ml*h) amongst the c.521T/C and c.521T/T genotypes. Gray circles denote participants with the c.521T/C genotype (n=12). Black circles denote participants with the c.521T/T genotype (n=7). Red circles denote "high exposure subgroup" participants (c.521T/T, n=5; c.521T/C, n=1).

Supplemental Table S1. Pharmacokinetic parameters amongst the entire cohort.

	AVA	AVL	2-OH AVA	2-OH AVL
Cmax (ng/ml)	2.2 (1.6-3.5)	0.9 (0.7-1.2)	0.7 (0.5-1.0)	1.1 (0.8-1.6)
AUC_{0-24} (ng/ml*h)	17.1 (11.5-19.7)	8.7 (4.0-15.0)	6.4 (4.8-9.4)	14.5 (5.5-24.1)

Data expressed as median (IQR).

Supplemental Table S2. Correlations between demographic parameters and systemic exposure

	AVA	AVL	2-OH AVA	2-OH AVL
Age (yr)	0.10 (0.60)	-0.44 (0.02)	0.33 (0.09)	-0.41 (0.03)
Height (cm)	-0.07 (0.73)	-0.61 (<0.001)	0.10 (0.60)	-0.60 (<0.001)
Height (Z-Score)	-0.22 (0.23)	0.07 (0.72)	-0.33 (0.09)	0.06 (0.74)
Weight (kg)	0.09 (0.63)	-0.68 (<0.001)	0.10 (0.59)	-0.81 (<0.0001)
Weight (Z-score)	0.20 (0.33)	-0.39 (0.04)	0.03 (0.89)	-0.57 (0.001)
BMI (kg/m^2)	0.14 (0.53)	-0.57 (0.001)	0.09 (0.65)	-0.73 (<0.0001)
BMI (Z-score)	0.28 (0.14)	-0.28 (0.15)	0.12 (0.54)	-0.48 (0.009)

Data expressed as correlation coefficient R values and (p-values).

Supplement Table S3. Atorvastatin and atorvastatin analyte parameters stratified by demographic and developmental parameters

	AVA AUC_{0-24} (ng/ml*h)	AVL AUC_{0-24} (ng/ml*h)	2-OH AVA AUC_{0-24} (ng/ml*h)	2-OH AVL AUC_{0-24} (ng/ml*h)
**Gender**				
Female	15.7 (± 6.0)	12.1 (± 9.3)	7.3 (± 2.8)	18.0 (± 12.1)
Male	17.1 (± 8.7)	10.7 (± 9.5)	7.0 (± 3.5)	15.7 (± 13.2)
p value	0.56	0.76	0.56	0.53
**Ethnicity**				
White, non-Hispanic	15.0 (± 6.1)	10.0 (± 7.7)	6.9 (± 2.7)	16.4 (± 12.9)
White, Hispanic	18.7 (± 9.4)	13.2 (± 11.9)	7.6 (± 4.0)	17.2 (± 13.8)
African-American	14.0 (± 0.7)	11.4 (± 1.1)	6.1 (± 0.1)	16.7 (± 2.7)
p value	0.26	0.72	0.96	0.89
**Tanner**				
Breast/Testicular				
Stage 1	N/A	N/A	N/A	N/A
Stage 2	17.9 (± 11.2)	23.3 (± 11.5)	6.0 (± 2.0)	31.9 (± 13.5)
Stage 3	19.1	17.3	6.0	23.4
Stage 4	9.2 (± 5.6)	7.9 (± 6.2)	4.7 (± 2.1)	13.4 (± 10.6)
Stage 5	17.5 (± 6.2)	8.5 (± 6.5)	8.1 (± 3.3)	12.9 (± 9.9)
p value	0.20	0.04	0.13	0.05
Pubic				
Stage 1	8.5	15.4	5.1	39.2

Stage 2	23.0 (± 8.5)	27.7 (± 7.9)	6.7 (± 1.9)	32.7 (± 11.1)
Stage 3	8.1	7.8	4.1	12.7
Stage 4	6.4 (± 1.3)	5.0 (± 2.5)	3.6 (± 0.1)	8.5 (± 5.8)
Stage 5	17.5 (± 6.0)	8.9 (± 6.6)	8.0 (± 3.3)	13.7 (± 10.2)
p value	0.02	0.02	0.04	0.05

All data expressed as mean (± SD); Kruskal-Wallis test used for all analyses.
